# Supplementary material for: Modeling glioblastoma heterogeneity as a dynamic network of cell states
Source: Mol Syst Biol. 2021 Sep 16;17(9):e10105. doi: 10.15252/msb.202010105 (PMC8444284; doi:10.15252/msb.202010105)
Supplement: Supplementary file 5 — Source Data for Figure 3 [file MSB-17-e10105-s001.zip › Figure3A_sourcedata/GSEA_3065/nosigdb_state1.GseaPreranked.1622718409535/RICHARDS_ZHONG_NATURE_2018_OPC_UPREG.html]

Details for gene set RICHARDS\_ZHONG\_NATURE\_2018\_OPC\_UPREG[GSEA]

|  || Dataset | state1 |
| Phenotype | NoPhenotypeAvailable |
| Upregulated in class | na\_neg |
| GeneSet | RICHARDS\_ZHONG\_NATURE\_2018\_OPC\_UPREG |
| Enrichment Score (ES) | -0.81293195 |
| Normalized Enrichment Score (NES) | -2.6731873 |
| Nominal p-value | 0.0 |
| FDR q-value | 0.0 |
| FWER p-Value | 0.0 |
Table: GSEA Results Summary

  

Fig 1: Enrichment plot: RICHARDS\_ZHONG\_NATURE\_2018\_OPC\_UPREG      
 Profile of the Running ES Score & Positions of GeneSet Members on the Rank Ordered List

  

| PROBE | GENE SYMBOL | GENE\_TITLE | RANK IN GENE LIST | RANK METRIC SCORE | RUNNING ES | CORE ENRICHMENT || 1 | DBI |  |  | 110 | 0.352 | 0.0028 | No |
| 2 | RHOC |  |  | 316 | 0.256 | -0.0079 | No |
| 3 | ARL4A |  |  | 497 | 0.206 | -0.0181 | No |
| 4 | ANXA5 |  |  | 709 | 0.171 | -0.0328 | No |
| 5 | CNTN1 |  |  | 911 | 0.143 | -0.0476 | No |
| 6 | CD82 |  |  | 1024 | 0.131 | -0.0538 | No |
| 7 | DUSP6 |  |  | 1401 | 0.095 | -0.0884 | No |
| 8 | CA10 |  |  | 1674 | 0.077 | -0.1130 | No |
| 9 | LIMA1 |  |  | 1678 | 0.077 | -0.1103 | No |
| 10 | ETV5 |  |  | 3894 | 0.006 | -0.3359 | No |
| 11 | PSAT1 |  |  | 4318 | -0.001 | -0.3790 | No |
| 12 | SPRY4 |  |  | 6504 | -0.039 | -0.6002 | No |
| 13 | ARC |  |  | 6612 | -0.041 | -0.6095 | No |
| 14 | MMP2 |  |  | 7051 | -0.051 | -0.6521 | No |
| 15 | RAB31 |  |  | 7256 | -0.057 | -0.6706 | No |
| 16 | CNP |  |  | 7303 | -0.058 | -0.6730 | No |
| 17 | RAMP1 |  |  | 7655 | -0.068 | -0.7061 | No |
| 18 | NXPH1 |  |  | 7920 | -0.078 | -0.7299 | No |
| 19 | GNG7 |  |  | 8050 | -0.083 | -0.7398 | No |
| 20 | EPB41L2 |  |  | 8402 | -0.099 | -0.7716 | No |
| 21 | LMF1 |  |  | 8699 | -0.117 | -0.7971 | No |
| 22 | SERPINE2 |  |  | 8736 | -0.120 | -0.7960 | No |
| 23 | TRIB2 |  |  | 8853 | -0.129 | -0.8027 | No |
| 24 | MT3 |  |  | 8954 | -0.139 | -0.8074 | Yes |
| 25 | C2orf80 |  |  | 8993 | -0.142 | -0.8056 | Yes |
| 26 | FIBIN |  |  | 9036 | -0.145 | -0.8041 | Yes |
| 27 | GATM |  |  | 9049 | -0.147 | -0.7995 | Yes |
| 28 | CD63 |  |  | 9126 | -0.154 | -0.8011 | Yes |
| 29 | CADM2 |  |  | 9141 | -0.157 | -0.7963 | Yes |
| 30 | ANGPTL2 |  |  | 9201 | -0.166 | -0.7958 | Yes |
| 31 | KANK1 |  |  | 9229 | -0.169 | -0.7918 | Yes |
| 32 | EPN2 |  |  | 9249 | -0.172 | -0.7869 | Yes |
| 33 | SIRT2 |  |  | 9386 | -0.198 | -0.7929 | Yes |
| 34 | RFTN2 |  |  | 9402 | -0.200 | -0.7864 | Yes |
| 35 | SOX8 |  |  | 9456 | -0.215 | -0.7833 | Yes |
| 36 | PLEKHB1 |  |  | 9466 | -0.218 | -0.7756 | Yes |
| 37 | PDE4B |  |  | 9522 | -0.231 | -0.7720 | Yes |
| 38 | SPRY1 |  |  | 9540 | -0.236 | -0.7643 | Yes |
| 39 | CST3 |  |  | 9581 | -0.254 | -0.7583 | Yes |
| 40 | SEZ6L |  |  | 9628 | -0.272 | -0.7522 | Yes |
| 41 | SPATA6 |  |  | 9657 | -0.286 | -0.7437 | Yes |
| 42 | TSC22D4 |  |  | 9677 | -0.296 | -0.7339 | Yes |
| 43 | LRRC4C |  |  | 9682 | -0.299 | -0.7224 | Yes |
| 44 | BCHE |  |  | 9684 | -0.300 | -0.7106 | Yes |
| 45 | SLC35F1 |  |  | 9715 | -0.327 | -0.7006 | Yes |
| 46 | CSPG5 |  |  | 9721 | -0.330 | -0.6880 | Yes |
| 47 | SPARC |  |  | 9738 | -0.347 | -0.6759 | Yes |
| 48 | ITM2C |  |  | 9758 | -0.363 | -0.6634 | Yes |
| 49 | ETV1 |  |  | 9764 | -0.371 | -0.6492 | Yes |
| 50 | OLIG2 |  |  | 9776 | -0.386 | -0.6349 | Yes |
| 51 | B2M |  |  | 9791 | -0.408 | -0.6201 | Yes |
| 52 | ITGB8 |  |  | 9794 | -0.418 | -0.6037 | Yes |
| 53 | PDGFRA |  |  | 9806 | -0.440 | -0.5874 | Yes |
| 54 | LSAMP |  |  | 9814 | -0.481 | -0.5690 | Yes |
| 55 | ALCAM |  |  | 9818 | -0.493 | -0.5497 | Yes |
| 56 | S100B |  |  | 9822 | -0.507 | -0.5298 | Yes |
| 57 | EDNRB |  |  | 9824 | -0.509 | -0.5097 | Yes |
| 58 | CD9 |  |  | 9832 | -0.544 | -0.4888 | Yes |
| 59 | PLAT |  |  | 9833 | -0.550 | -0.4669 | Yes |
| 60 | PON2 |  |  | 9835 | -0.554 | -0.4450 | Yes |
| 61 | SCD5 |  |  | 9838 | -0.576 | -0.4224 | Yes |
| 62 | OLIG1 |  |  | 9845 | -0.637 | -0.3977 | Yes |
| 63 | NOVA1 |  |  | 9858 | -0.741 | -0.3694 | Yes |
| 64 | SEMA5A |  |  | 9860 | -0.749 | -0.3398 | Yes |
| 65 | SOX6 |  |  | 9863 | -0.798 | -0.3083 | Yes |
| 66 | PTPRZ1 |  |  | 9869 | -0.918 | -0.2723 | Yes |
| 67 | PMP2 |  |  | 9871 | -0.982 | -0.2334 | Yes |
| 68 | TTYH1 |  |  | 9872 | -1.033 | -0.1923 | Yes |
| 69 | GPM6B |  |  | 9873 | -1.091 | -0.1489 | Yes |
| 70 | PTN |  |  | 9874 | -1.107 | -0.1049 | Yes |
| 71 | BCAN |  |  | 9878 | -1.175 | -0.0585 | Yes |
| 72 | SCRG1 |  |  | 9879 | -1.473 | 0.0000 | Yes |
Table: GSEA details [plain text format]

  

Fig 2: RICHARDS\_ZHONG\_NATURE\_2018\_OPC\_UPREG: Random ES distribution      
 Gene set null distribution of ES for **RICHARDS\_ZHONG\_NATURE\_2018\_OPC\_UPREG**
